# Inhibition of CDK9 sensitizes multidrug resistant ovarian cancer cells to paclitaxel

**DOI:** 10.1038/s41598-026-47843-6

**Published:** 2026-04-07

**Authors:** Jinglu Wang, Francis J. Hornicek, Huirong Shi, Wenlong Feng, Zhenfeng Duan

**Affiliations:** 1 Department of Obstetrics and Gynecology, The First Affiliated Hospital of Zhengzhou University, 1 Jianshe East Road, Zhengzhou, 450052 Henan China; 2 Department of Orthopedic Surgery, Sarcoma Biology Laboratory, Sylvester Comprehensive Cancer Center, University of Miami Miller School of Medicine, Papanicolaou Cancer Research Building, 1550 NW. 10th Avenue, Miami, FL 33136 USA; 3 Department of Orthopedic Surgery, Sylvester Comprehensive Cancer Center, University of Miami Miller School of Medicine, United States of American, Papanicolaou Cancer Research Building, 1550 NW. 10th Avenue, Miami, FL 33136 USA

**Keywords:** Ovarian cancer, CDK9, LDC000067/LDC067, Drug resistance, Transcription, Apoptosis, Cancer, Cell biology, Drug discovery, Oncology

## Abstract

Multidrug resistance is the major cause of treatment failure in ovarian cancer. Overexpression of cyclin-dependent kinase (CDK) 9 has been observed in a variety of cancers and promotes tumor cell growth and proliferation by regulating transcription. However, the role of CDK9 in drug resistance of ovarian cancer remains unknown. In this study, the expression levels of CDK9 and related proteins were evaluated in two pairs of human ovarian cancer drug-resistant cell lines by western blot. We found that both drug resistant ovarian cancer cells expressed higher levels of CDK9 as compared with their parental drug sensitive cell lines. Additionally, inhibition of CDK9, with either siRNA or a CDK9 inhibitor LDC067, enhanced paclitaxel sensitivity in drug resistant ovarian cancer cells. This was also accompanied with a reduction in the phosphorylation of RNA polymerase II and Stat3, resulting in enhanced apoptosis. Furthermore, inhibition of CDK9 also decreased ovarian cancer spheroid formation and cell motility. These findings collectively indicate that inhibition of CDK9 can enhance paclitaxel sensitivity in drug resistant ovarian cancer cells by increasing apoptosis. CDK9 represents a promising target for overcome drug resistance in ovarian cancer.

**Supplementary Information:**

The online version contains supplementary material available at 10.1038/s41598-026-47843-6.

## Introduction

Ovarian cancer is the sixth-leading cause of cancer death among women and the deadliest of gynecologic cancers because it is often diagnosed at an advanced stage^1^. The American Cancer Society estimates that approximately 20,890 new ovarian cancer cases and 12,730 deaths will occur in the United States in 2025 alone^1^. Despite the advances in surgical techniques and chemotherapy drugs for several decades, the overall 5-year survival rate of ovarian cancer patients increased slightly from 36% in 1975 to 51% in 2020^1,2^. Currently, the microtubule stabilizing based chemotherapy agent paclitaxel (trade name Taxol) is widely used in the clinical treatment of ovarian cancer in combination with cisplatin (or carboplatin). However, most ovarian cancer patients who are initially responsive to paclitaxel will inevitably develop broad cross resistance (multidrug resistance, MDR) that can include a variety of structurally and functionally unrelated chemotherapeutic agents^3^. MDR is a major clinical problem that severely limits the success rate of chemotherapy in ovarian cancer treatment, leading to patient relapse or even death due to drug resistant/metastatic disease^4^. Therefore, therapeutic exploitation of more potent agents to sensitize MDR cells to chemotherapy is essential to improve the clinical outcome of ovarian cancer.

Cyclin-dependent kinases (CDKs) are members of the serine/threonine family of protein kinases and play important roles in cell cycle progression and RNA transcription^5–7^. In many cancers, CDKs have been found to be overexpressed and highly activated, rendering it as an attractive therapeutic target^8,9^. Over the past three decades, CDK inhibitors have been developed for the treatment of numerous cancers, including breast cancer and leukemia^10–12^. The first cyclin CDK4/6 inhibitor palbociclib (Ibrance, Pfizer) has been approved by the U.S. FDA in 2015 as a first-line treatment of ER positive, HER2 negative advanced breast cancer^11^.

Mammalian cells contain 21 different CDKs, but only a few subsets of CDK–cyclin complexes are directly associated with cell-cycle progression or RNA transcription. Among these CDKs, CDK9 appears to be one of the most important. CDK9 and cyclin T complex, which is a component of the positive transcription elongation factor b (P-TEFb), promotes transcription elongation via phosphorylation of RNA polymerase II (RNAPII)^13,14^. Overexpression and activation of CDK9 have been reported in various tumors, including breast cancer, prostate cancer, gastric cancer, lymphoma, pancreatic cancer, oral squamous cell carcinoma and sarcoma^15–22^. Increased CDK9 expression is associated with aggressive clinicopathological features and worse prognosis. CDK9 is emerging as a promising therapeutic target in cancer treatment and several CDK9 inhibitors have already undergone evaluation in clinical trials^23^. Our previous study demonstrates that CDK9 is highly expressed in metastatic and recurrent ovarian cancer tissues and correlates with poor prognosis^24^. Inhibition of CDK9 can enhance the sensitivity of tumor cells to chemotherapeutic drugs has been observed in several types of tumors^19,25,26^. However, the role of CDK9 in drug resistance of ovarian cancer remains controversial unknown. The purpose of this study is to investigate the effects of CDK9 inhibitor alone or in combination with paclitaxel in drug resistant ovarian cancer cells in vitro as well as its potential underlying mechanisms.

## Results

### CDK9 expression level is correlated with drug resistance in ovarian cancer cell lines

**Fig. 1:**
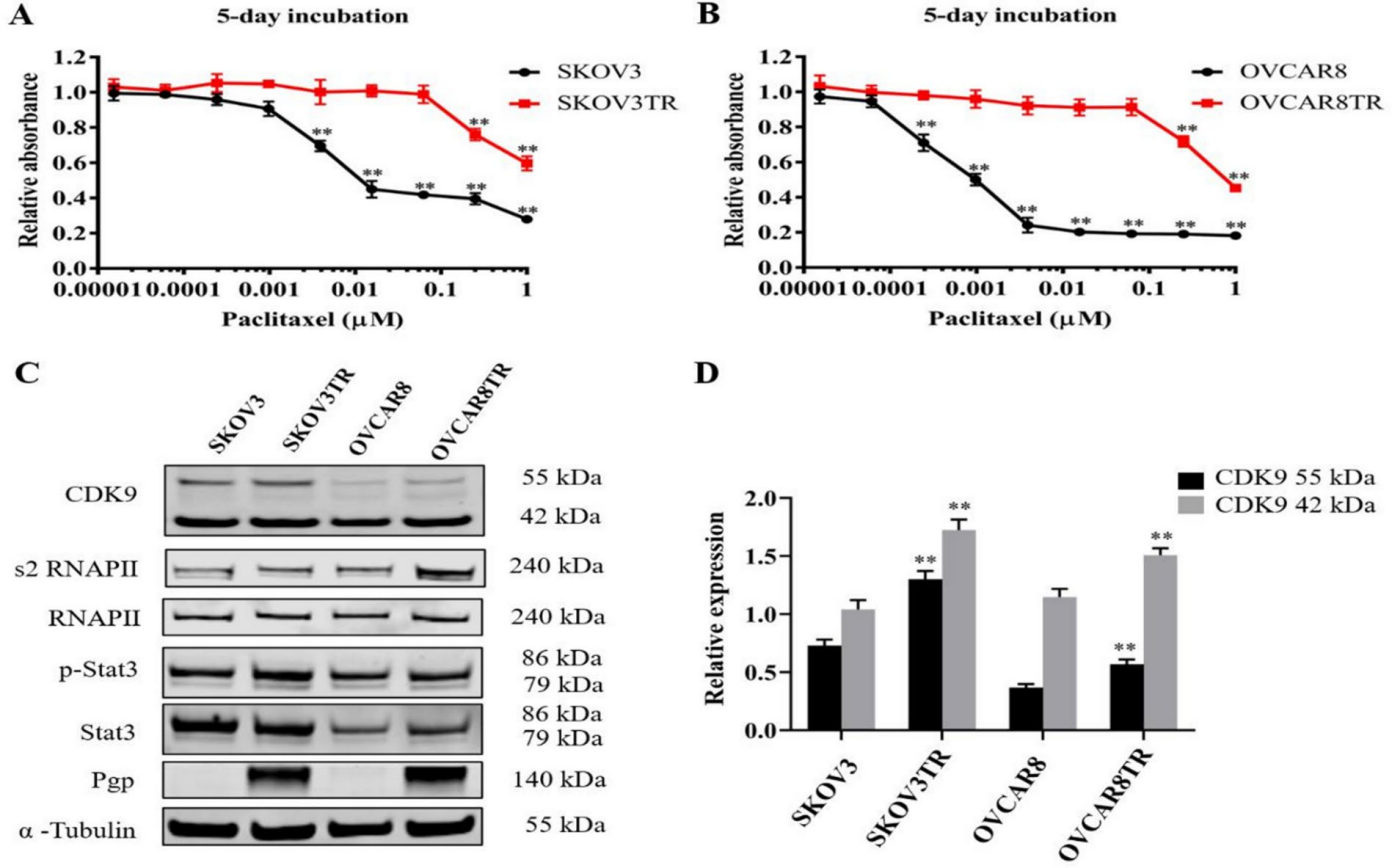
Higher expression of CDK9 was present in the resistant ovarian cancer cell line compared to the parental sensitive cell line. (**A**) Relative cell viability of SKOV3 and SKOV3TR cells after incubation with different concentration of paclitaxel for five days. (**B**) Relative cell viability of OVCAR8 and OVCAR8TR cells after incubation with different concentration of paclitaxel for five days. (**C**) Expression levels of CDK9 and related signaling pathway proteins involved in transcription in SKOV3TR and OVCAR8TR, and parental sensitive cell lines SKOV3 and OVCAR8 were determined by Western blot. There are 2 isoforms of the CDK9 protein: the 42 kDa CDK9 isoform and the 55 kDa isoform. The smaller 42 kDa isoform was the first identified isoform. The larger, 55 kDa isoform has a characteristic 117 residue terminal expansion. (**D**) Relative expression of both CDK9 isoforms and α-Tubulin in the ovarian cancer cell lines. **P < 0.01.

In our previous study, we demonstrated that CDK9 is highly expressed in metastatic and recurrent ovarian cancer tissues, and correlates with poor prognosis^24^. Based on the results, we further examined the expression of CDK9 in two pairs of drug sensitive and their resistant ovarian cancer cell lines SKOV3/SKOV3TR and OVCAR8/OVCAR8TR. As observed in the MTT assay, SKOV3TR and OVCAR8TR cells exhibited stable growth in culture medium containing 0.3 µM of paclitaxel, while SKOV3 and OVCAR8 cells were unable to survive in a culture medium supplemented with 0.03 µM of paclitaxel (Fig. 1A and B). This result indicated that SKOV3TR and OVCAR8TR cells were resistant to paclitaxel whereas SKOV3 and OVCAR8 cells were sensitive to paclitaxel. Based on western blot results, MDR cell lines SKOV3TR and OVCAR8TR exhibited higher expression of P-glycoprotein (Pgp) than their parental sensitive cell lines. CDK9 is expressed in two isoforms; a lighter 42 kDa isoform, and a heavier 55 kDa isoform which is transcribed from an upstream transcriptional start site which extends the shared mRNA sequence. This larger 55KD protein has an additional 117 amino acids at the N-terminus^27^. Western blot analysis revealed SKOV3TR and OVCAR8TR expressed significantly higher levels of CDK9 compared with parental sensitive cell lines. Moreover, phosphorylated RNAPII (s2) and Stat3 (Tyr705) expressed higher in SKOV3TR and OVCAR8TR than parental sensitive cell lines, while there was no statistical significant of RNAPII and Stat3 (Fig. 1C and D, Fig. S1).

### CDK9 silencing suppresses RNAPII phosphorylation and enhances drug sensitivity in vitro

**Fig. 2:**
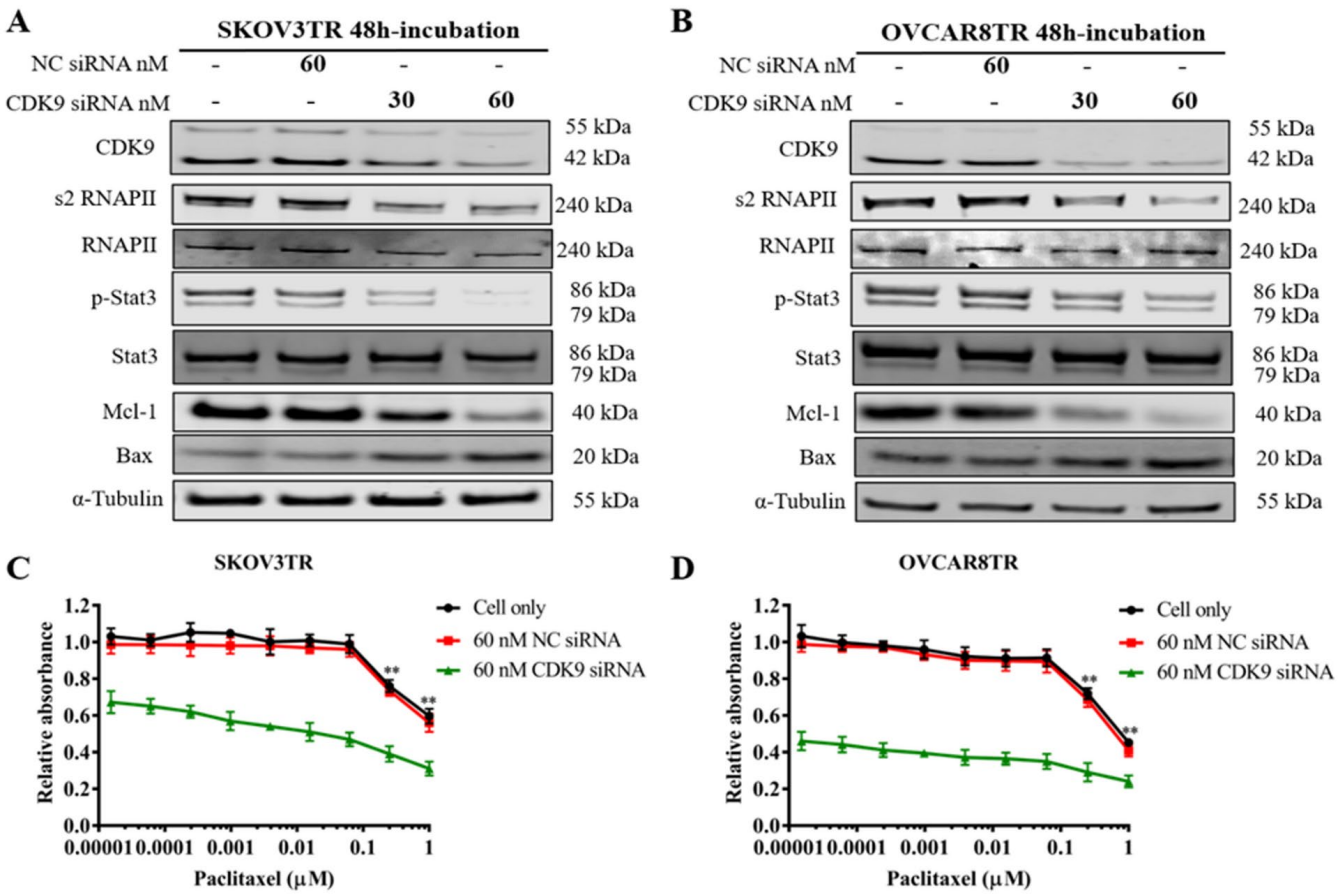
CDK9 knockdown by siRNA transfection enhanced paclitaxel sensitivity in resistant ovarian cancer cells. (**A** and **B**) Expression levels of CDK9 and related signaling pathway proteins involved in transcription and apoptosis after transfection of CDK9 siRNA and nonspecific siRNA in SKOV3TR and OVCAR8TR cell lines by Western blot. (**C** and **D**) MTT assay revealed enhanced sensitivity to paclitaxel of SKOV3TR and OVCAR8TR cells after CDK9 siRNA transfection. **P < 0.01 indicates that the observed results of CDK9 knockdown by siRNA transfection enhanced paclitaxel sensitivity in drug resistant SKOV3TR or OVCAR8TR cell lines are highly statistically significant as compared with the same non-transfected cell lines or nonspecific siRNA transfected cell lines.

To evaluate whether suppression of CDK9 affects the growth and drug sensitivity of ovarian cancer cells, we transfected both SKOV3TR and OVCAR8TR cells with CDK9 siRNA to knock down CDK9 expression. Western blot showed that CDK9 expression was markedly inhibited by 30 nM CDK9 siRNA, whereas cells transfected with nonspecific siRNA expressed a constant level of CDK9 and other proteins compared to untreated cells (Fig. 2A and B). The expression of RNAPII was not altered after CDK9 knockdown, however, the phosphorylated RNAPII (s2) expression levels were significantly downregulated in SKOV3TR and OVCAR8TR cells. Knockdown of CDK9 also resulted in decreased levels of phosphorylated Stat3 (Tyr705) and antiapoptotic protein Mcl-1, as well as increased level of the proapoptotic protein Bax (Fig. 2A and B, Fig. S2). In addition, as compared to untreated and nonspecific siRNA transfected cells, the sensitivity to paclitaxel was enhanced when CDK9 was knocked down (Fig. 2C and D). Taken together, these results suggest that CDK9 may play a critical role in drug resistance of ovarian cancer cells.

### CDK9 inhibitor has a synergistic effect with paclitaxel on anti-proliferation and sensitize drug resistant ovarian cancer cells to paclitaxel

**Fig. 3:**
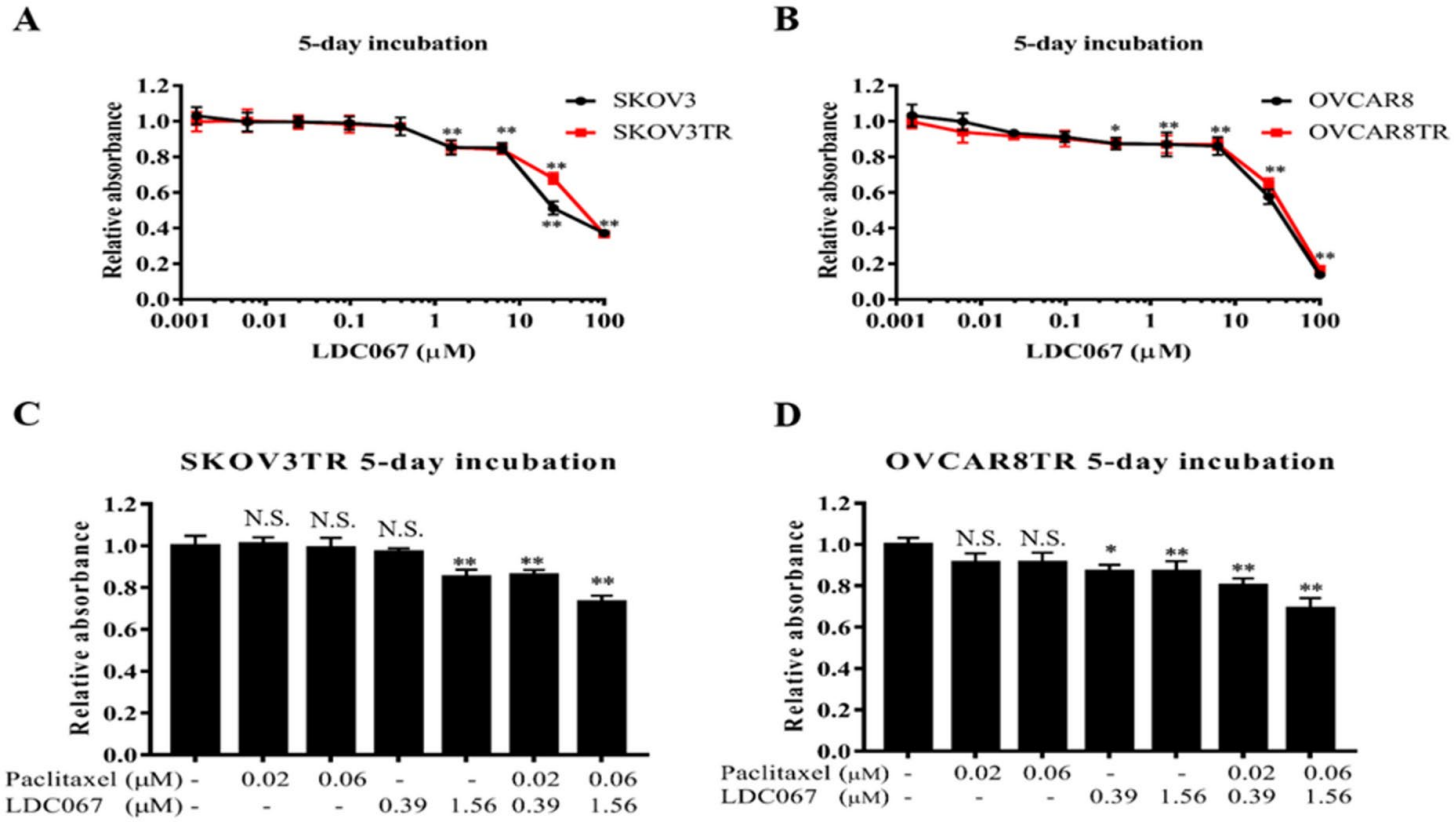
Effects of combination treatment with paclitaxel and CDK9 inhibitor LDC067 on the viability of multidrug resistant ovarian cancer cell line. (**A**) Relative cell viability of SKOV3 and SKOV3TR cells after incubation with different concentration of CDK9 inhibitor LDC067 for five days. (**B**) Relative cell viability of OVCAR8 and OVCAR8TR cells after incubation with different concentration of CDK9 inhibitor LDC067 for five days. (**C** and **D**) Relative cell viability of SKOV3TR and OVCAR8TR cells in combination with paclitaxel and CDK9 inhibitor LDC067 for five days. N.S. indicates that the observed data for paclitaxel and LDC067 alone treated groups are not statistically significant as compared with untreated groups; *P < 0.05, the observed data for paclitaxel and LDC067 combinatorial treated groups are statistically significant as compared with the untreated groups; **P < 0.01, the observed results for paclitaxel and LDC067 combinatorial treated groups are highly statistically significant as compared with the untreated groups.

The SKOV3/SKOV3TR and OVCAR8/OVCAR8TR cells were exposed to increasing concentration of paclitaxel with or without different concentration of CDK9 inhibitor (LDC067) to further evaluate whether suppression of CDK9 affects the growth and drug sensitivity of ovarian cancer cells. Figure 3A and B showed that no significant difference was observed in the sensitivity to CDK9 inhibitor between the paired sensitive and resistant cell lines, suggesting that unlike paclitaxel, LDC067 itself is not a substrate of Pgp. CDK9 inhibitor suppresses cell proliferation in a dose-dependent manner in SKOV3TR and OVCAR8TR cells (Fig. 3A and B). Next, we assessed the combination effect of paclitaxel and CDK9 inhibitor. Figure 3C and D showed that, paclitaxel and CDK9 inhibitor combinations have an increased antiproliferative effect compared with either agent alone, and the inhibition effect indicated a significant does-dependent manner. The peak synergistic effect was noted with the combination of 0.02 µM paclitaxel + 0.39 µM LDC067, 1 µM paclitaxel + 25 µM LDC067 (Table 1). When these MDR cells were treated with combination of 1 µM paclitaxel + 25 µM LDC067, the SKOV3TR and OVCAR8TR survival rates decreased to 40% of the control groups, respectively. These data indicate that, when combined with CDK9 inhibitor, the response to paclitaxel is increased in drug resistant ovarian cancer cells.

**Table 1:** Effect of paclitaxel and LDC067 combination treatment in SKOV3TR and OVCAR8TR cell lines according to the Chou and Talalay method^28^.

Cell lines	Schedule Paclitaxel+LDC067	FA	CI	Effect
SKOV3TR	0.02 µM + 0.39 µM	0.14	0.17	Strongly synergistic
	0.06 µM + 1.56 µM	0.27	0.22	Strongly synergistic
	0.25µM + 6.25µM	0.35	0.52	Synergistic
	1 µM + 25µM	0.76	0.19	Strongly synergistic
OVCAR8TR	0.02 µM + 0.39 µM	0.20	0.28	Strongly synergistic
	0.06 µM + 1.56 µM	0.31	0.36	Synergistic
	0.25µM + 6.25µM	0.42	0.57	Synergistic
	1 µM + 25µM	0.72	0.20	Strongly synergistic

Note: FA means the growth effect of drug-treated cells compared with control cells and CI denotes the combination index. CI > 1.1 indicates antagonism; 0.9–1.1 indicates additive; 0.7–0.9 indicates moderate synergism; 0.3–0.7 indicates synergism; <0.3 indicates strong synergism.

### CDK9 inhibitor suppresses the CDK9 downstream pathways in drug resistant ovarian cancer cells

**Fig. 4:**
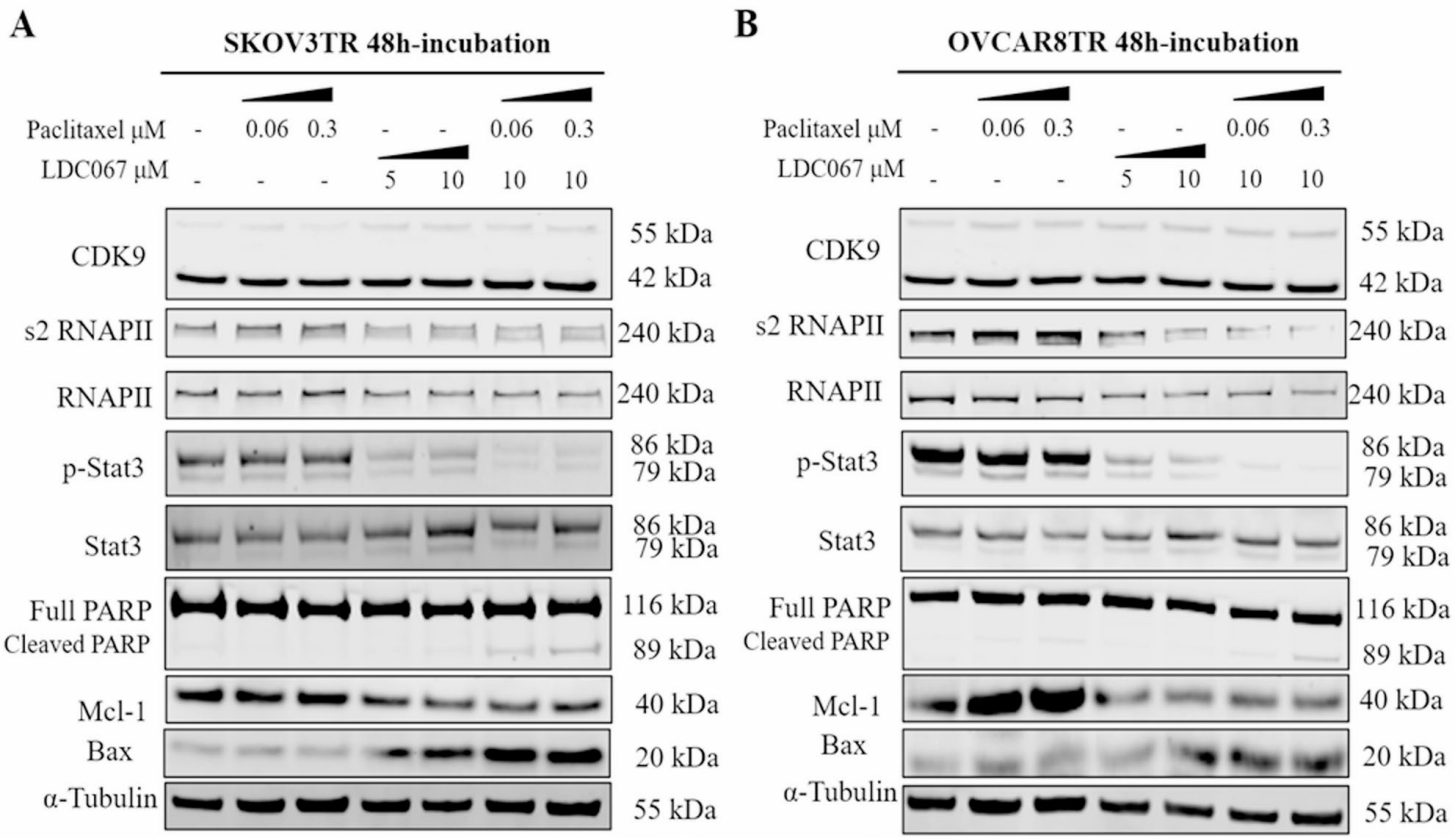
Effects of treatment with paclitaxel and CDK9 inhibitor LDC067 on the transcription and apoptosis in multidrug resistant ovarian cancer cell line. (**A** and **B**) Expression levels of CDK9 and related signaling pathway proteins involved in transcription and apoptosis after treatment with paclitaxel and CDK9 inhibitor LDC067 in SKOV3TR and OVCAR8TR cell lines by Western blot.

To further characterize the mechanisms of CDK9 inhibition on transcription regulation in drug resistant ovarian cancer cells, western blot analysis was performed to examine the effects of paclitaxel alone, LDC067 alone, and their combination on CDK9 downstream pathways in SKOV3TR and OVCAR8TR cells, including RNA polymerase II (RNAPII), Stat3, and apoptosis-related proteins. Due to resistant to paclitaxel, there was no apparent influence on CDK9 downstream pathways proteins when SKOV3TR and OVCAR8TR cells were treated with paclitaxel alone (Fig. 4A and B). Treatment with the CDK9 inhibitor significantly suppressed CDK9 downstream pathway expression in a dose-dependent manner. Incubation of SKOV3TR and OVACAR8TR cells with 5 and 10 µM of LDC067 for 48 h showed a gradual reduction of phosphorylated RNAPII (S2) and p-Stat3 without a significant change in overall RNAPII and Stat3 expression. Similar to siRNA exposure, in CDK9 inhibitor treated cells, the antiapoptotic protein Mcl-1 decreased, and the proapoptotic protein Bax increased in a dose-dependent manner. As expected, there was no apparent influence on CDK9 expression, as the CDK9 inhibitor LDC067 functionally inhibits CDK9 activity but not its overall expression. Next, we assessed the combination effect of paclitaxel and CDK9 inhibitor LDC067. The SKOV3TR and OVACAR8TR cells were treated with paclitaxel alone or in the combination with LDC067. Combination treatment resulted in synergistic effects as compared with LDC067 or paclitaxel treatment alone. Moreover, combination treatment showed a significant increase of pro-apoptotic proteins cleaved PARP expression (Fig. 4A and B). Overall, these data suggest that CDK9 inhibitor suppresses the CDK9 downstream pathways in drug resistant ovarian cancer cells and combination treatment with paclitaxel increases the inhibitory effect.

### Inhibition of CDK9 suppresses spheroid growth and clonogenicity formation in drug resistant ovarian cancer cells

**Fig. 5:**
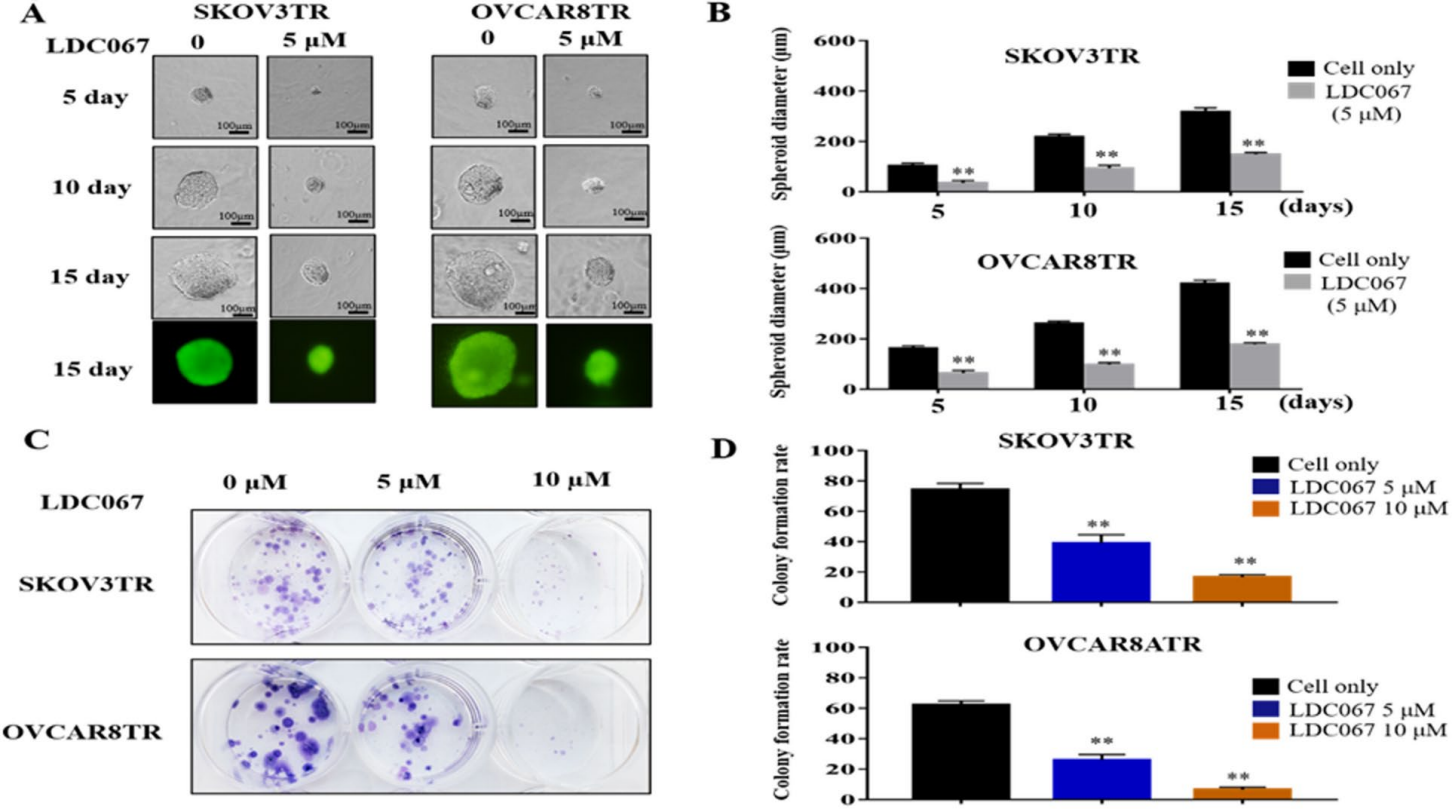
Inhibition of CDK9 suppressed drug resistant ovarian cancer cell spheroid growth, and clonogenicity formation. (**A**) Representative images of drug resistant ovarian cancer cells SKOV3TR and OVCAR8TR after treatment with the CDK9 inhibitor LDC067 at different time points (5 days, 10 days, and 15 days). (**B**) The relative diameters of spheroids in SKOV3TR and OVCAR8TR cells after LDC067 treatment. **P < 0.01 compared with the cell only group. (**C**) Representative images of drug resistant ovarian cancer cells SKOV3TR and OVCAR8TR colony formation after incubation with different concentrations of LDC067 (0, 5, 10 µM) for 14 days. (**D**) Quantification of clonogenicity formation of SKOV3TR and OVCAR8TR cells after LDC067 treatment. **P < 0.01 compared with the cell only group.

3D culture simulates the in vivo environment for cancer cells by permitting the cells to form their natural 3D spheroid shape. The SKOV3TR and OVACAR8TR cells were exposed to 5 µM LDC067 for a period of 15 days in 3D culture and formed spheroids were photographed across multiple time points. Although the diameter of cell spheroids without CDK9 inhibitor treatment continuously grew, the spheroids formed from LDC067 treated cells were significantly smaller than the untreated cells (Fig. 5A and B). After 15 days of drug treatment, SKOV3TR and OVCAR8TR spheroids showed the growth of diameters were 148.67 μm and 178.67 μm, while the untreated group reached to 317.67 μm and 420 μm respectively. To further determine the effect of CDK9 inhibition on clonogenicity of SKOV3TR and OVACAR8TR cells, we treated these cells with 5 µM and 10 µM of LDC067 for 14 days. The colony formation assay demonstrated that LDC067 exposure caused a dose-dependent reduction in the number and size of colonies formed compared to the untreated cells (Fig. 5C and D). Under treatment with LDC067 at 5 µM and 10 µM, SKOV3TR cells showed colony formation rates of 39% and 16.67% respectively, compared to 74.33% in the untreated control group. Similarly, OVCAR8TR cells exhibited colony formation rates of 26% and 6.67% when treated with 5 µM and 10 µM LDC067, compared to 62.33% in the untreated group. These results support the role of CDK9 in enhancing the growth and progression of drug resistant ovarian cancer cells.

### Inhibition of CDK9 reduces cell migration in drug resistant ovarian cancer cells

**Fig. 6:**
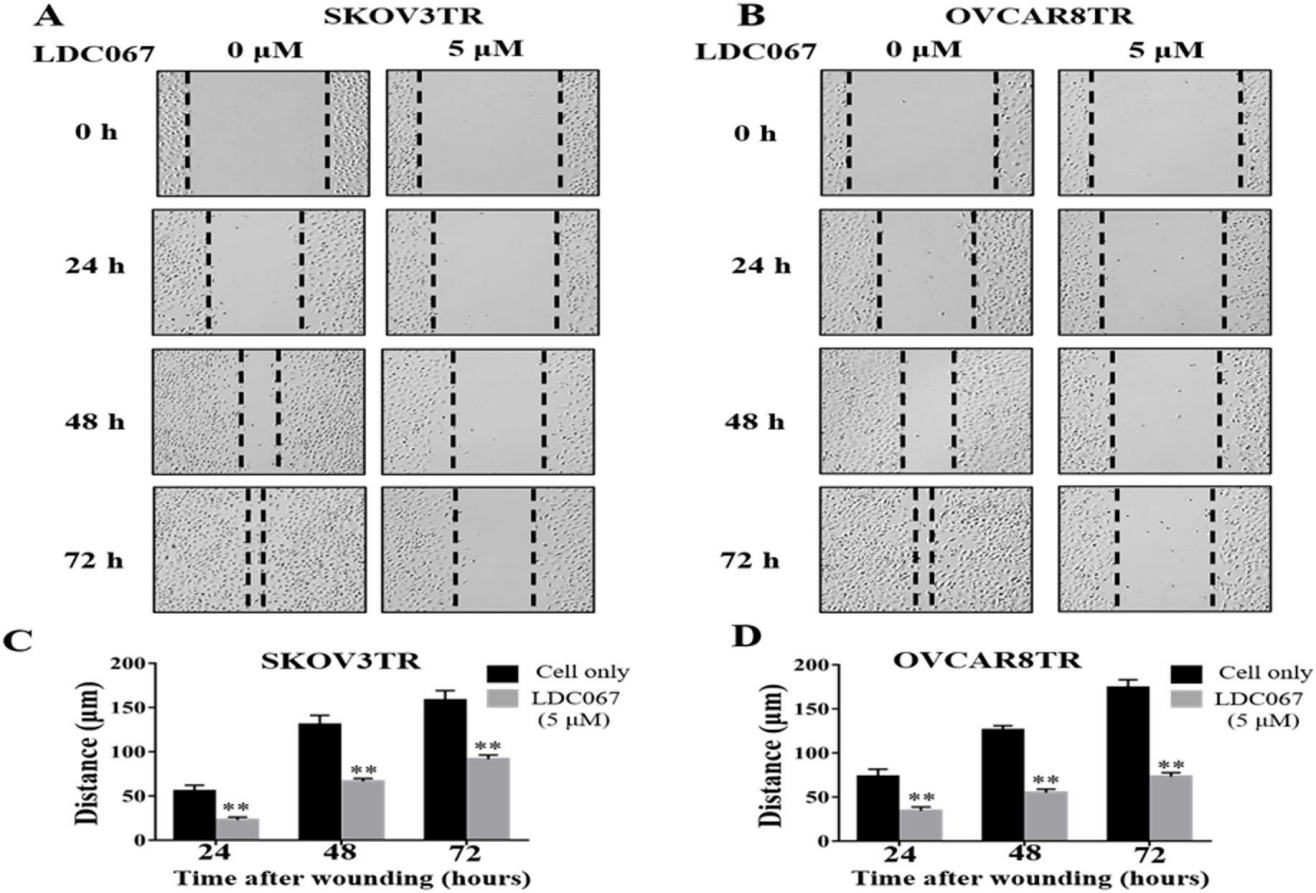
Effect of treatment with CDK9 inhibitor LDC067 on the migratory activity of multidrug resistant ovarian cancer cell line. (**A** and **B**) Relative migration distance of SKOV3TR and OVCAR8TR cells at different time points (0 h, 24 h, 48 h, 72 h) when treated with the CDK9 inhibitor LDC067. (**C** and **D**) Quantification of cell migration distance of SKOV3TR and OVCAR8TR cells after LDC067 treatment. **P < 0.01 compared with the cell only group.

Migration of ovarian cancer cells is crucial prerequisite for cancer metastasis and recurrence. As previous study showed that CDK9 expression significantly correlated with the metastasis and recurrence of ovarian cancer patients, we further evaluated the role of CDK9 in the modulation of cell mobility in vitro. The wound healing assay was performed in drug resistant ovarian cancer cell lines after CDK9 inhibitor treatment. As illustrated in Fig. 6A and B, the addition of 5 µM of LDC067 for 24, 48, and 72 h led to a time-dependent decrease in cell migration in both cell lines compared to the untreated cells (*P* < 0.05). After 72-hours of LDC067 incubation, SKOV3TR and OVCAR8TR migrated a total of 91 μm and 73 μm, while the untreated cells migrated 158 μm and 174 μm respectively (Fig. 6C and D). The results demonstrated that inhibition of CDK9 suppressed the migration capabilities of drug resistant ovarian cancer cells.

## Discussion

The development of drug resistance is a primary reason for the recurrence of ovarian cancer. In clinic, the vast majority of patients initially respond to chemotherapy treatment but eventually relapse due to the acquired drug resistance of tumor cells. Resistance emerges through various mechanisms, including the overexpression of ATP-binding cassette (ABC) transporter based membrane protein Pgp, and activation of survival pathways. Overexpression of CDK9 promotes cell proliferation and the formation of anti-apoptotic factors, such as MCL-1, BCL-2, and XIAP, which are critical factors for the survival of cancer cells^26,29^. Our previous study showed that both metastatic and recurrent ovarian cancer patient tissues expressed higher levels of CDK9 when compared with the matched primary tumor samples^24^. Similar correlations of high CDK9 expression and poor clinical features were also found in ovarian cancer and other cancer types such as osteosarcoma and chordoma^9,15,30^. Moreover, knockdown of CDK9 with siRNA and inhibition of CDK9 activity with inhibitor suppressed RNA transcription elongation, induced apoptosis, and reduced proliferation of cancer cells^9,15,31^. Recent studies also showed that CDK9 may plays a role in the development of drug resistance. The involvement of CDK9 to drug resistance is primarily due to its function as a central transcriptional regulator of genes that drive cancer cell survival, proliferation, and anti-apoptotic signaling^21,22,32^.

To investigate the role of CDK9 in the development of drug resistance in ovarian cancer, western blot analysis was applied to compare parental and established paclitaxel-resistant ovarian cancer cells. We observed that the drug resistant ovarian cancer cells expressed higher levels of CDK9, s2 RNAPII, and p-Stat3 as compared with their parental drug sensitive cell lines. CDK9 protein has two known isoforms, both isoforms have been shown to be highly expressed in MDR cell lines. Collectively, these results suggest that aberrant CDK9 expression may be associated with the development of drug resistance.

In our study, we found that siRNA mediated CDK9 knockdown restored the sensitivity of drug resistant ovarian cancer cells to paclitaxel. Targeting CDK9 has been shown to resensitize resistant cancer cells thus enhance cytotoxicity of chemotherapeutic drugs. Studies have showed CDK9 inhibitors can be effective against drug-resistant cancers, including Osimertinib-resistant non-small cell lung cancer (NSCLC), chemotherapy-resistant small cell lung cancer (SCLC), and multiple myeloma (MM) resistant to other therapies^21,22,32^. Some cancers may develop resistance by altering pathways related to cell cycle regulation or by activating apoptosis associated survival signaling events. CDK9 inhibitors can counteract these mechanisms, restoring sensitivity to therapy^22,33^. Our current study showed combination treatment with the CDK9 inhibitor and paclitaxel has a strong synergistic effect on anti-proliferation and sensitization of MDR ovarian cancer cells to paclitaxel. The combination of paclitaxel and CDK9 inhibitor had an enhanced antiproliferative effect when compared with either agent alone. In previous study, CDK9 inhibition potently enhances the therapeutic effect of chemotherapeutics in various cancer cells^19,25,26^. CDK9 inhibitor SNS-032 synergizes with the anticancer drug irinotecan in pancreatic cancer cells, reducing cell viability by approximately 40% compared to monotherapy^19^. Combination of CDK9 inhibitor SNS-032 and Ara-C increases the sensitivity of acute myeloid leukemia (AML) cells to the cytotoxic effects of Ara-C by inhibiting the transcription of antiapoptotic genes^26^. Another CDK9 inhibitor, CDKI-73, effectively induced apoptosis as a single agent and synergized anti-tumor activity of cisplatin in hypopharyngeal carcinoma cells^25^. A phase 2 clinical trial study revealed combined CDK inhibitor flavopiridol and cisplatin therapy has activity in platin-resistant and platin-sensitive ovarian cancer with acceptable toxicities^34^. These studies support our findings that the combination of CDK9 inhibitor with traditional chemotherapeutic agents is a promising treatment option for ovarian cancer, and its therapeutic effect can be evaluated in further clinical trials for ovarian cancer.

Several CDK9 inhibitors including LDC067 have been in preclinical studies, some of them have already been into clinical trials^35–39^. In this study, we evaluated the novel selective CDK9 inhibitor LDC067, which has been used in studies in various types of malignancies^9,15,31,40^. To uncover the molecular mechanism(s) by which LDC067 inhibits the growth of ovarian cancer cells, we found that phosphorylation of RNAPII and Stat3 was significantly higher in SKOV3TR and OVCAR8TR than parental sensitive cell lines. Prior research has demonstrated that Stat3 not only promotes the initiation of transcription but also regulates chromatin remodeling and transcription elongation through its interaction with CDK9^41,42^. Drug resistant recurrent tumors have significantly greater phosphorylated Stat3 expression as compared with matched primary tumors. Inhibition of Stat3 activation results in significant decreases in paclitaxel resistance and enhanced apoptosis^43^. We found that inhibition of CDK9 by small interfering RNA or CDK9 inhibitor LDC067 suppressed RNA transcription elongation and Stat3 activation, and reduced proliferation of drug resistant ovarian cancer cells. Gene expression profiling of cells treated with LDC067 demonstrated a selective reduction of short-lived mRNAs, including key regulators of proliferation and apoptosis such as Mcl-1 and Myc^40^. The nuclear enzyme PARP plays a well-established role in base excision and single strand DNA break repair. According to previous reports, the action of paclitaxel is mediated by downregulation of Bcl-xL and activation of PARP, resulting in apoptosis induction^44^. Our results showed that upregulation of PARP cleavage and Bax, downregulation of Mcl-1 expression were indicative of increased apoptosis after LDC067 and paclitaxel co-treatment. Therefore, we conclude that apoptosis induction serves as a key mechanism underlying the effect of the LDC067/paclitaxel treatment combination in drug resistant ovarian cancer cells. The combined treatment may overcome MDR in these cells.

Aberrations of cell motility and migration are fundamental drivers of cancer metastasis and recurrence. Tumor cells exploit and alter normal mechanisms of cell movement to break away from a primary tumor, travel to distant sites, and establish new colonies^45^. Considering that the TMA results of ovarian cancer patients showed that CDK9 was significantly correlated with metastasis and recurrence, we investigated the role of CDK9 in the modulation of cell mobility *in vitro*^24^. Inhibition of CDK9 enhances the migration and invasive ability of drug resistant ovarian cancer cells. 3D cell culture is an artificially created condition that mimics the *in vivo* environment, in which cells are permitted to grow or interact with their surroundings in three dimensions. We found that CDK9 inhibition prevents ovarian cancer cells spheroid formation and also decreases the speed of cell growth. Furthermore, inhibition of CDK9 also suppresses the clonogenic survival and migratory capabilities of drug resistant ovarian cancer cells. Collectively, these results indicate that CDK9 may be a contributor to ovarian cancer metastasis.

## Conclusions

In conclusion, we demonstrate that inhibition of CDK9 by inhibitor LDC067 or a CDK9-specific siRNA can increase the sensitivity to paclitaxel in drug resistant ovarian cancer cells. The underlying mechanisms of this phenomenon are in part through decreased RNAPII phosphorylation and Stat3 activation, resulting in enhanced apoptosis (i.e., upregulated PARP cleavage and Bax expression, downregulated Mcl-1 expression). Our results of supporting therapeutic potential of targeting CDK9 to overcome MDR in patients with ovarian cancer.

## Materials and methods

### Cell lines and reagents

Previously, we have characterized and described the ovarian cancer drug sensitive cell lines SKOV3 and OVCAR8, and their MDR derivatives SKOV3TR and OVCAR8TR that were used in this study^46–48^. Both SKOV3TR and OVCAR8TR were established from their respective parental cell lines by exposure to stepwise increases in paclitaxel concentrations. The resistant derivatives were found to be 100-fold more resistant to paclitaxel compared to the sensitive parental cell lines^47^. These cell lines were cultured in RPMI 1640 (GE Healthcare Life Sciences, Logan, Utah, USA) and supplemented with 10% fetal bovine serum (Sigma-Aldrich, St. Louis, MO, USA) with 1% penicillin/streptomycin (Life Technologies, Carlsbad, CA, USA) at 37°C in a 5% CO2 humidified atmosphere. The human ovarian cancer cell line SKOV3 was purchased from the American Type Culture Collection (Rockville, MD) in 2014 with certificate of analysis. Dr. Patricia Donahoe (Massachusetts General Hospital, Boston, MA) kindly provided the human ovarian cancer cell line OVCAR8^49^. Both the cell lines have been widely used in different ovarian cancer research as showed in various publications. The highly selective CDK9 inhibitor LDC000067 (abbreviated to LDC067) was purchased from Selleck Chemicals (#S7461, Houston, Texas, USA). Paclitaxel was obtained as previously described^46^. The anticancer drug paclitaxel was obtained from Teva Pharmaceuticals (Sellersville, PA, USA). Human nonspecific siRNA and CDK9 targeting siRNA (5’-GCUGCUAAUGUGCUUAUCA-3’) were purchased from Sigma-Aldrich. Lipofectamine^®^ RNAiMAX was purchased from Thermo Fisher Scientific (Waltham, MA, USA). The monoclonal rabbit anti-human CDK9 antibody was purchased from Cell Signaling Technology (Danvers, MA, USA). The RNAPII associated antibodies, including RNAPII and phosphorylated RNAPII (S2), were purchased from Abcam (Cambridge, MA, USA). Apoptosis related antibodies and monoclonal rabbit anti-human Pgp antibody were obtained from Cell Signaling Technology. The Stat3 pathway associated antibodies, including Stat3 and phosphorylated Stat3 (Tyr705) were also purchased from Cell Signaling Technology.

### Work flow of Lipofectamine-mediated transfection of CDK9 siRNA

Knockdown of CDK9 in ovarian cells was performed by transfection of synthetic CDK9 siRNA. In brief, ovarian cancer cells were seeded into 96-well plates at a density of 2 × 10^3^ cells per well or into 12-well plates at a density of 6 × 10^4^ cells per well and transfected with 30 nM, and 60 nM of synthesized CDK9 siRNA using the Lipofectamine RNAiMax reagent (Thermo Fisher Scientific) according to the manufacturer’s instructions. Nonspecific siRNA (60 nM) was used as a negative control. The experimental methods have been described in our previous studies^24^.

### Methyl thiazolyl tetrazolium (MTT) assay

The cytotoxicities of CDK9 inhibitor LDC000067 (LDC067) and paclitaxel in ovarian cancer cells were assessed by MTT (3(4,5-dimethylthiazol-2-yl)-2,5-diphenyl tetrazolium bromide) assay. In brief, 3 × 10^3^ cells per well were seeded into a 96-well microplate and exposed to different concentrations of LDC067 with or without paclitaxel. After a 5-day incubation, 20 µl MTT (Sigma-Aldrich) was added after which the cells were incubated for 4 h at 37 °C in a 5% CO_2_ humidified atmosphere. Subsequently, the resulting intracellular formazan crystals were solubilized in acid-isopropanol. The absorbances were assessed using a SpectraMax Microplate^®^ Spectrophotometer (Molecular Devices LLC, Sunnyvale, CA, USA) at 490 nm, and normalized to those of untreated cells. In all cases, the MTT assay was conducted in triplicate.

### Synergistic effect assay

The effect of the combination of paclitaxel and CDK9 inhibitor (LDC067) on cell proliferation was assessed by calculating combination index (CI) values using Compusyn Software (Biosoft). Derived from the median-effect equation of Chou and Talalay, the CI provides a quantitative measure for synergism, additive effect or antagonism in drug combination^28^. CI over 1.1 indicates antagonism, 0.9–1.1 additive, 0.7–0.9 moderate synergism, 0.3–0.7 synergism, and < 0.3 strong synergism.

### Western blot analysis

Protein lysates of ovarian cancer cell lines were extracted using 1 × RIPA lysis buffer (Sigma-Aldrich) supplemented with complete protease inhibitor cocktail tablets (Roche Applied Science, Indianapolis, IN, USA). Protein concentrations were calculated by the Protein Assay Reagents (Bio-Rad, Hercules, CA, USA) and a SpectraMax 340PC Microplate Reader from Molecular Devices (San Jose, CA, USA). Equal amounts of proteins were separated by NuPAGE^®^ 4–12% Bis-Tris Gel (Thermo Fisher Scientific) and subsequently transferred to a nitrocellulose membrane (Bio-Rad), which was then incubated with specific primary antibodies at 4 °C overnight: CDK9 (Cell Signaling Technology, Cat.#2316, 1:1000 dilution); RNAPII (Abcam, Cat.#ab817, 1:1000 dilution), S2 RNAPII (Abcam, Cat.#ab5095, 1:1000 dilution); Pgp (Cell Signaling Technology, Cat.#9532, 1:1000 dilution); PARP (Cell Signaling Technology, Cat.#13342, 1:1000 dilution); Stat3 (Cell Signaling Technology, Cat.#9139, 1:1000 dilution); Phospho-Stat3 (Cell Signaling Technology, Cat.#9145, 1:1000 dilution); Mcl-1 (Cell Signaling Technology, Cat.#39224, 1:1000 dilution); Bax (Cell Signaling Technology, Cat.#2772, 1:1000 dilution); α -Tubulin (Cell Signaling Technology, Cat.#3873, 1:1000 dilution). After being washed three times for 5 min with TBST, the membranes were further incubated with Goat anti-rabbit IRDye 800CW or Goat anti-mouse IRDye 680LT secondary antibody (LI-COR Biosciences, Lincoln, NE, USA), and scanned by Odyssey^®^ CLx equipment (LI-COR Biosciences) to detect the bands. Finally, protein bands were quantified by densitometry using the Odyssey 3.0 software.

### Three-dimensional (3D) cell culture assay

In order to mimic the *in vivo* environment, a 3D cell culture assay was applied to assess the effect of CDK9 on cell growth. Hydrogel of ovarian cancer cell lines was established in 24-well VitroGel™ 3D cell culture plates with a density of 2 × 10^4^ cells/well, according to the manufacturer’s protocol (TheWell Bioscience Inc., Newark, NJ, USA). Immediately following this, different cell culture media (with or without 5 µM of LDC067) was added to cover the hydrogel. The well plate was then placed in an incubator, and its cover media was changed every 48 h to provide sufficient nutrients for the cells. The spheroid formations were photographed with a Nikon microscope (Diagnostic Instruments Inc.) every 5 days. At the end of the 15-day period, cell culture media containing 0.25 µM Calcein AM (Invitrogen) was added to cover the hydrogel. After 15 min of incubation, the spheroids were imaged on the Nikon Eclipse Ti-U fluorescence microscope equipped with a SPOT RT™ digital camera. The experimental methods have been described in our previous studies^24^.

### Clonogenic assay

Clonogenic assay was conducted to evaluate the effect of CDK9 on cell viability and proliferation. The ovarian cancer cell lines were seeded into 12-well plates at 100 cells/well and exposed to different concentrations of the CDK9 inhibitor LDC067 (0, 5, 10 µM). After a 14-day incubation period, the cells were fixed with methanol for 10 min, washed three times with PBS, then stained with 10% Giemsa stain (Sigma-Aldrich) for 20 min. Finally, the colonies were washed with flowing water and allowed to dry. Pictures of the stained colonies were captured using a digital camera (Olympus, Tokyo, Japan). The experimental methods have been described in our previous studies^24^.

### Wound healing assay

Cell migration activity was determined with a wound healing assay. In brief, ovarian cancer cells were seeded into 6-well plates at a density of 4 × 10^5^ cells per well and incubated overnight. The adherent cell layer was then scraped into two parallel lines with a sterile 30 µL tip. Immediately afterward, 5 µM of the CDK9 inhibitor LDC067 was added into the cell medium for an additional 72 h of starved incubation with a low-serum medium containing 3% FBS. After 0, 24, 48, and 72 h of applied LDC067 treatment, the wounds were photographed with a Nikon microscope (Diagnostic Instruments Inc., NY, USA) equipped with Zen Imaging software. The wound width was evaluated by measuring the distance between the two edges of the scratch at five sites in each image. Cell migration distance was determined with the following formula: (wound width at the 0 h time point - wound width at the observed time point) / 2. The experimental methods have been described in our previous studies^24^.

### Statistical analysis

Statistical analysis was performed using GraphPad Prism 10.0 software (Graph Pad Software Inc., San Diego, CA, USA). Independent two-tailed Student’s t-tests were performed to analyze the differences between two groups. In all cases, results were presented as the mean ± standard error of mean (SEM) and *P* values < 0.05 were considered statistically significant. All blots represent triple independent experiments.

## Supplementary Information

Below is the link to the electronic supplementary material.

Supplementary Material 1

Supplementary Material 2

Supplementary Material 3

Supplementary Material 4

## Data Availability

The date that support the findings of this study are available from the corresponding author upon reasonable request.

## Abbreviations

CDK9: cyclin-dependent kinase 9
MDR: multidrug resistance
LDC067: LDC000067
RNAPII: RNA polymerase II
MTT: Methyl thiazolyl tetrazolium
CI: combination index
3D: Three-dimensional (3D)

## Declarations

### Animal studies

N/A.
