# Supplementary figures and images for: Inhibition of CDK9 sensitizes multidrug resistant ovarian cancer cells to paclitaxel
Source: Sci Rep. 2026 Apr 7;16:11671. doi: 10.1038/s41598-026-47843-6 (PMC13062015; doi:10.1038/s41598-026-47843-6)

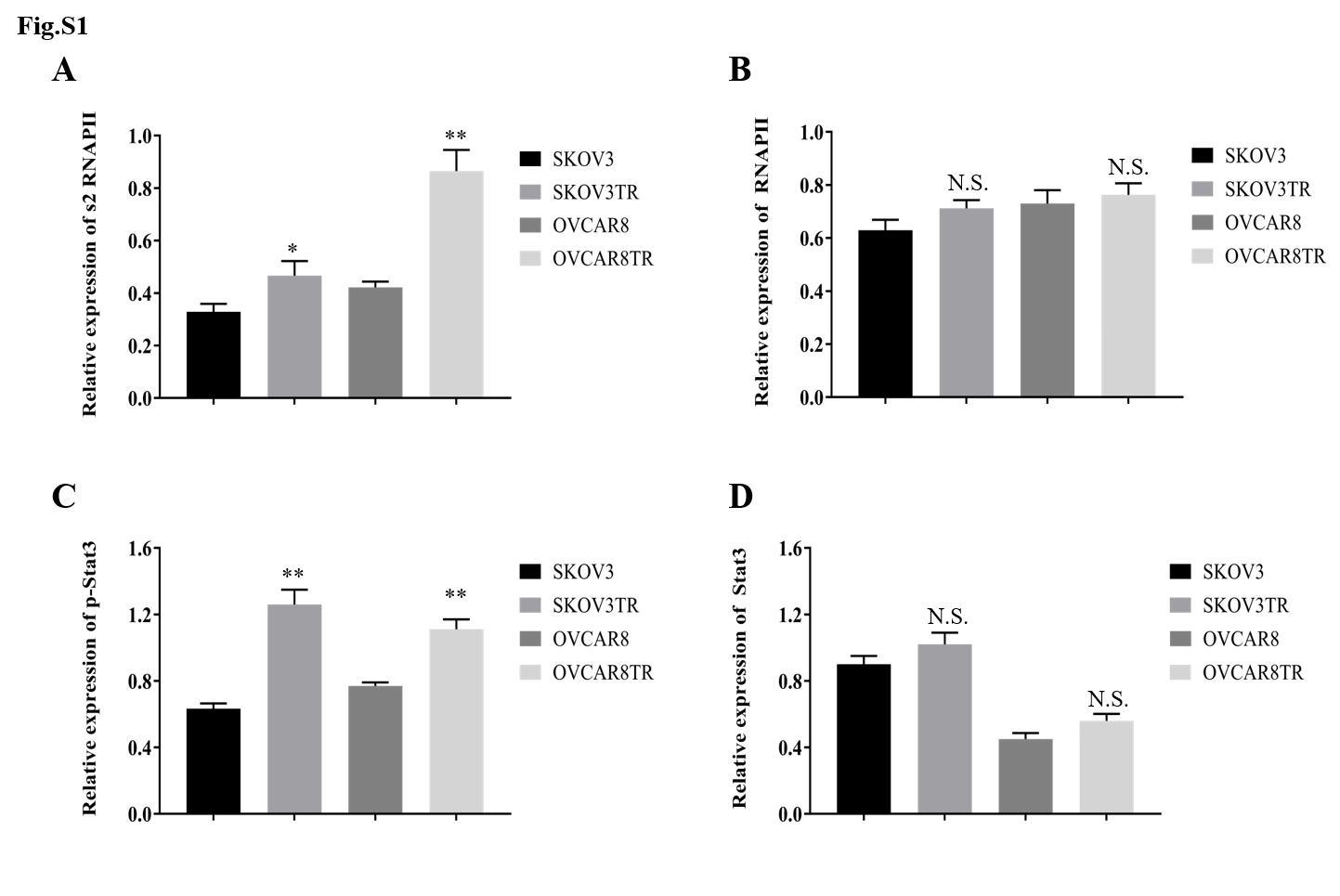

Supplement: Supplementary file 1 — Supplementary Material 1 [file 41598_2026_47843_MOESM1_ESM.tif]

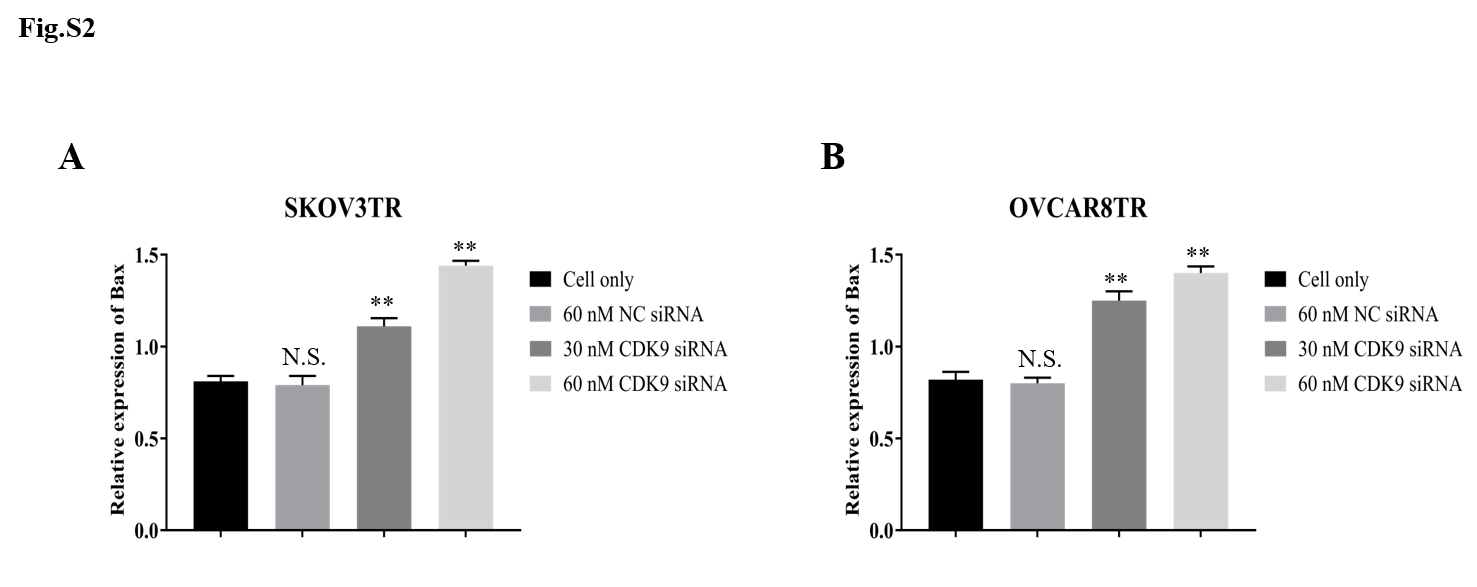

Supplement: Supplementary file 2 — Supplementary Material 2 [file 41598_2026_47843_MOESM2_ESM.tif]
