# Supplementary material for: Inhibition of CDK9 sensitizes multidrug resistant ovarian cancer cells to paclitaxel
Source: Sci Rep. 2026 Apr 7;16:11671. doi: 10.1038/s41598-026-47843-6 (PMC13062015; doi:10.1038/s41598-026-47843-6)
Supplement: Supplementary file 3 — Supplementary Material 3 [file 41598_2026_47843_MOESM3_ESM.pdf]

Figure 1C

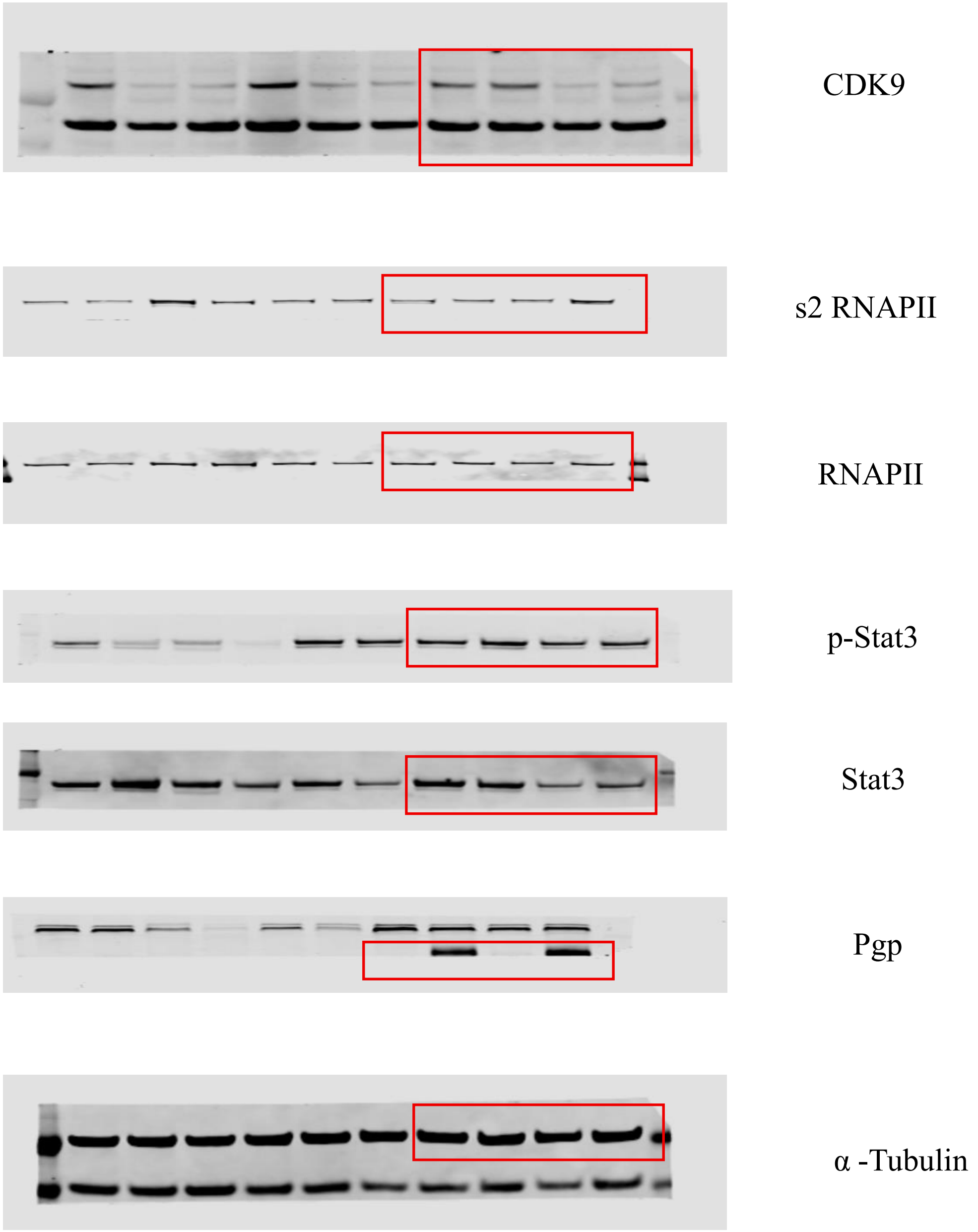

Figure 2A

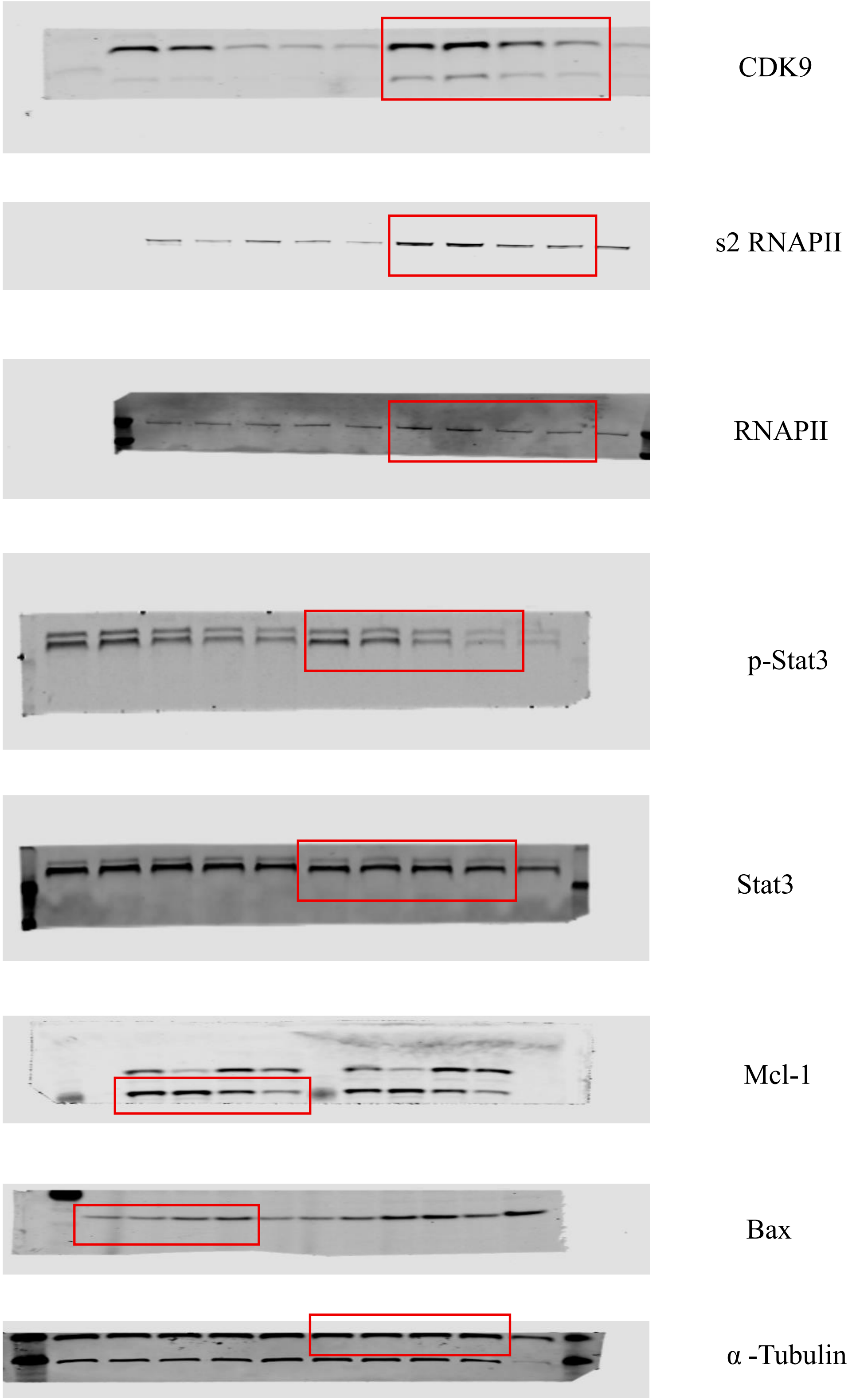

Figure 2B

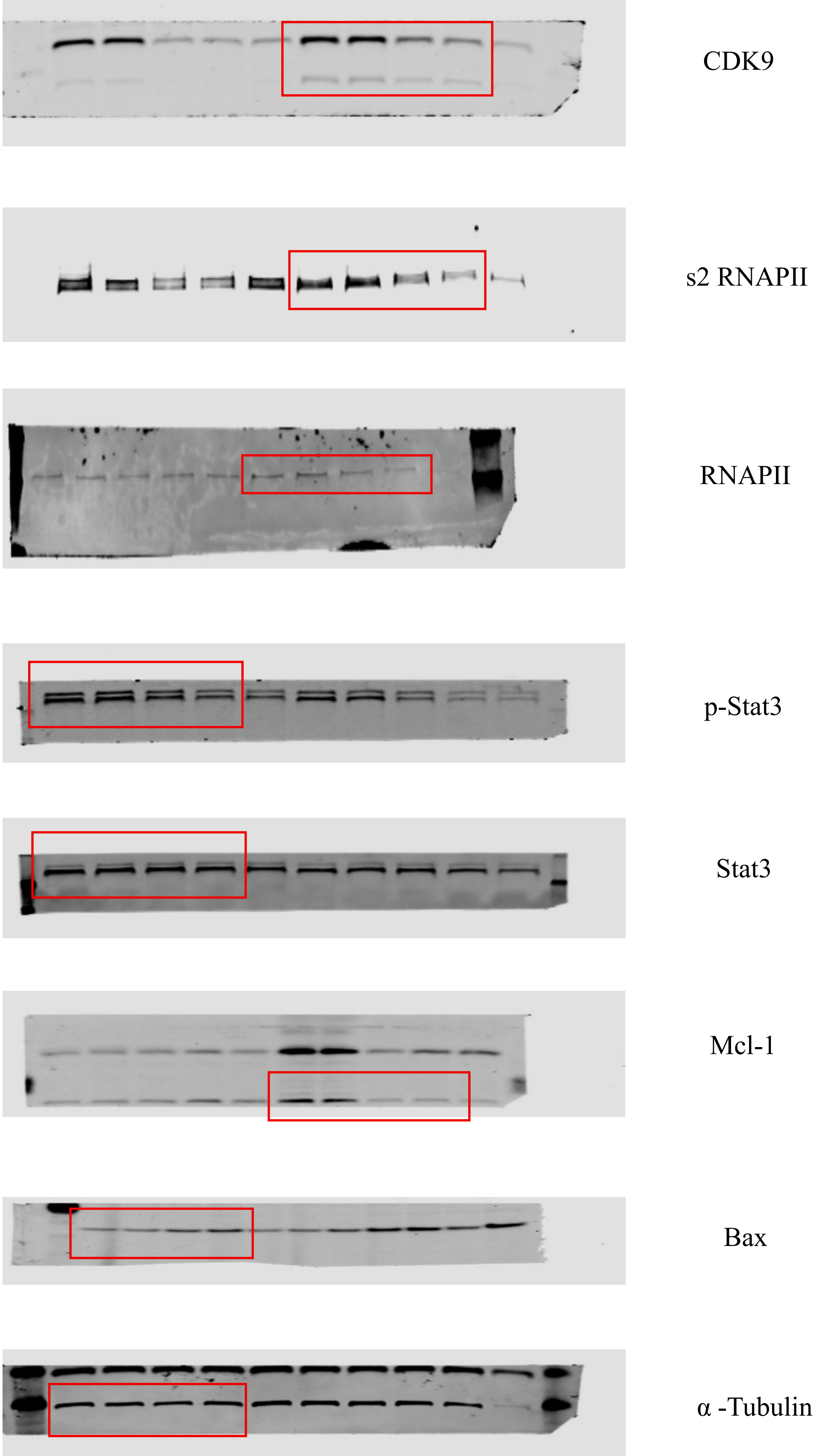

Figure 4A

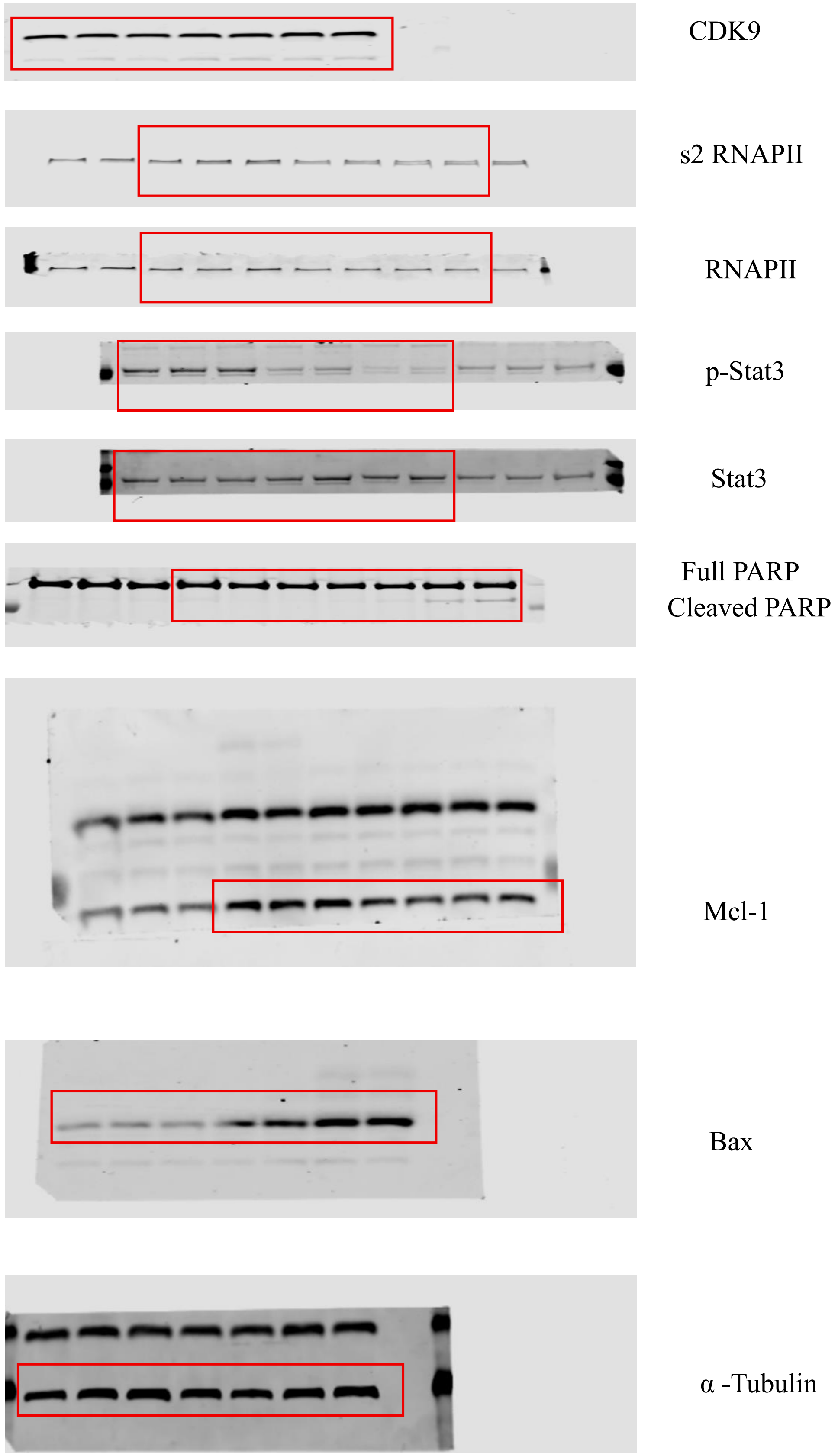
CDK9
s2 RNAPII
RNAPII
p-Stat3
Stat3
Full PARP
Cleaved PARP
Mcl-1
Bax
α -Tubulin

Figure 4B

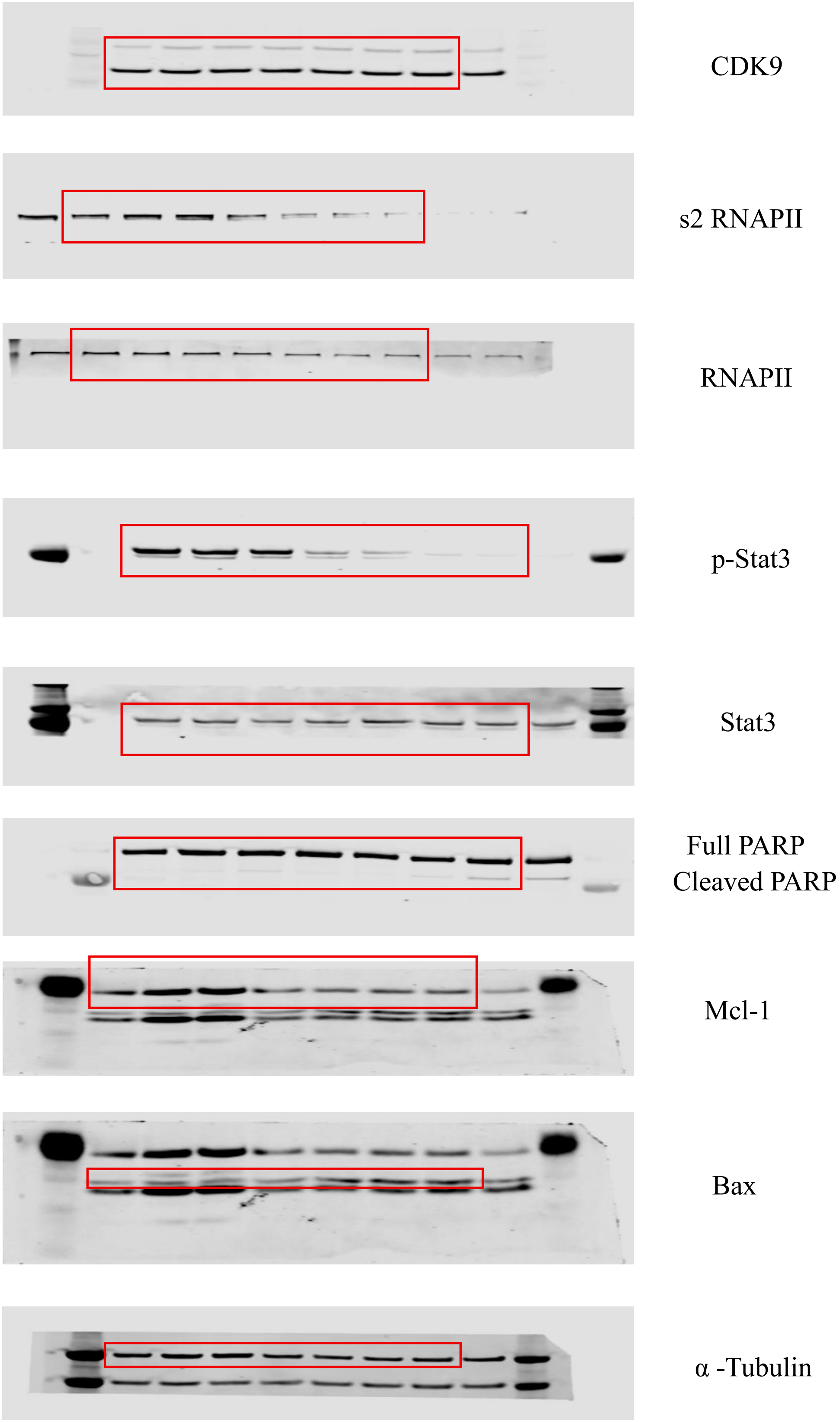
CDK9
s2 RNAPII
RNAPII
p-Stat3
Stat3
Full PARP
Cleaved PARP
Mcl-1
Bax
α -Tubulin
