# Supplementary material for: Inhibition of CDK9 sensitizes multidrug resistant ovarian cancer cells to paclitaxel
Source: Sci Rep. 2026 Apr 7;16:11671. doi: 10.1038/s41598-026-47843-6 (PMC13062015; doi:10.1038/s41598-026-47843-6)
Supplement: Supplementary file 4 — Supplementary Material 4 [file 41598_2026_47843_MOESM4_ESM.docx]

**Supplementary Figure Legends**

**Figure S1:** Relative expression of related signaling pathway proteins in the ovarian cancer cell lines. (A) Relative expression of s2 RNAPII and α-Tubulin in the ovarian cancer cell lines. (B) Relative expression of RNAPII and α-Tubulin in the ovarian cancer cell lines. (C) Relative expression of p-Stat3 and α-Tubulin in the ovarian cancer cell lines. (D) Relative expression of Stat3 and α-Tubulin in the ovarian cancer cell lines. N.S. indicates that the expression of related signaling pathway proteins are not statistically significant in SKOV3TR or OVCAR8TR drug resistant cell line as compared with SKOV3 or OVCAR8 drug sensitive cell line; **P*<0.05, the observed data for the expression of related signaling pathway proteins are statistically significant in SKOV3TR or OVCAR8TR drug resistant cell line as compared with SKOV3 or OVCAR8 drug sensitive cell line, respectively; ***P*<0.01, the observed data for the expression of related signaling pathway proteins are highly statistically significant in SKOV3TR or OVCAR8TR as compared with SKOV3 or OVCAR8, respectively.

**Figure S2:** Relative expression of Bax after transfection of CDK9 siRNA and nonspecific siRNA in the ovarian cancer cell lines. (A) Relative expression of Bax and α-Tubulin after transfection of CDK9 siRNA and nonspecific siRNA in SKOV3TR cell line. (B) Relative expression of Bax and α-Tubulin after transfection of CDK9 siRNA and nonspecific siRNA in OVCAR8TR cell line. N.S. indicates that the expression of Bax is not statistically significant in nonspecific siRNA-transfected SKOV3TR or OVCAR8TR drug-resistant cell lines as compared with the same non-transfected cell lines, respectively; ***P*<0.01, the observed data for the expression of Bax is highly statistically significant in CDK9 siRNA-transfected SKOV3TR or OVCAR8TR drug resistant cell lines as compared with the same non-transfected cell lines, respectively.
